# Co-Benefit Assessment of Active Transportation in Delhi, Estimating the Willingness to Use Nonmotorized Mode and Near-Roadway-Avoided PM_2.5_ Exposure

**DOI:** 10.3390/ijerph192214974

**Published:** 2022-11-14

**Authors:** Tavoos Hassan Bhat, Hooman Farzaneh, Nishat Tasnim Toosty

**Affiliations:** 1 Interdisciplinary Graduate School of Engineering Sciences, Kyushu University, Fukuoka 816-8580, Japan; 2 Transdisciplinary Research and Education Center for Green Technologies, Kyushu University, Fukuoka 816-8580, Japan; 3 Department of Statistics, University of Dhaka, Dhaka 1000, Bangladesh

**Keywords:** nonmotorized transport, health impact assessment, near-roadway PM_2.5_ exposure, physical activity

## Abstract

This study aims to estimate the avoided mortalities and morbidities and related economic impacts due to adopting the nonmotorized transportation (NMT) policy in Delhi, India. To this aim, an integrated quantitative assessment framework is developed to estimate the expected environmental, health, and economic co-benefits from replacing personal motorized transport with NMT in Delhi, taking into account the inhabitants’ willingness to use NMT (walking and cycling) mode. The willingness to accept NMT is estimated by conducting a cross-sectional survey in Delhi, which is further used to estimate the expected health benefits from both increased physical activity and near-roadway-avoided PM_2.5_ exposure in selected traffic areas in 11 major districts in Delhi. The value of a statistical life (VSL) and cost of illness methods are used to calculate the economic benefits of the avoided mortalities and morbidities from NMT in Delhi. The willingness assessment indicates that the average per capita time spent walking and cycling in Delhi is 11.054 and 2.255 min, respectively. The results from the application of the NMT in Delhi show the annual reduction in CO_2_ and PM_2.5_ to be 121.5 kilotons and 138.9 tons, respectively. The model estimates the expected co-benefits from increased physical activities and reduced PM_2.5_ exposure at 17,529 avoided cases of mortality with an associated savings of about USD 4870 million in Delhi.

## 1. Introduction

### 1.1. Physical Inactivity and Health Impacts

Physical inactivity and air pollution are two major public health issues currently being faced in India. Nonmotorized transport (NMT) especially walking and cycling, can reduce air pollution and physical inactivity, save lives, and lessen the effects of climate change. Physical inactivity is responsible for about 5 million annual deaths, while motorized transportation-related emissions are responsible for 74 thousand premature deaths, and Delhi has the highest transportation-related death rates among India’s major cities [1,2]. Sedentary lifestyles are one of the main factors increasing the risk of mortality from noncommunicable diseases (NCDs), as 71% of all deaths worldwide, 41 million per year, are caused by NCDs. Cardiovascular diseases (17.9 million), cancers (9.3 million), respiratory diseases (4.1 million), and diabetes (1.2 million) are major NCD-related mortalities each year [3]. Studies have shown that being physically inactive raises the risk of psychological disorders, colon cancer, breast cancer, type 2 diabetes, coronary heart disease, and musculoskeletal conditions by about 15% to 20% [4].

Physical activity (PA) has been shown in numerous studies to significantly reduce mortality. Being overweight, obesity, type 2 diabetes, coronary heart disease, depression, and fracture risk can all be decreased by PA. According to studies, sedentary lifestyles correspond to higher all-cause mortality [4]. About 1.6 million deaths have explicitly been linked to insufficient physical activity [3]. On a global scale, an increase in physical activity can prevent 3.9 million premature deaths, which equals 15% of all premature deaths [5]. It has been established that regular exercise can aid in preventing and treating NCDs, such as breast and colon cancer, diabetes, heart disease, and stroke. PA can also enhance mental health, quality of life, and well-being while preventing hypertension, obesity, and overweight [6,7,8]. Regardless of sex or age, PA is considered to have a preventative effect against depression and a beneficial impact on treating depression in nonclinical and clinical populations [9,10,11]. Walking for 30 min or bicycling for 20 min on most days lowers the mortality risk by at least 10%, also 10% reduction in the risk of cardiovascular disease and a 30% reduction in the risk of type 2 diabetes, as well as a 30% reduction in the mortality rate from cancer [12]. In India, 20% of the population is not active, and 37% is just moderately active, failing to meet the WHO physical activity guidelines, leading to the risk of developing various NCDs in a large portion of the Indian population [13]. The prevalence of NCDs in India, including breast and colon cancer, diabetes, coronary artery disease, and hypertension, are mainly related to physical inactivity [4]. Regular PA can help prevent and treat many NCDs [13].

### 1.2. Nonmotorized Transport in Delhi

The urban transportation system endangers human health through accidents, air pollution, and physical inactivity. Such health issues have demanded significant health impact analyses in order to facilitate the creation of walking and bicycling-friendly infrastructure to improve public health. India’s urbanization rate has led to substantial increases in the nation’s energy consumption, travel demand, and transportation-related emissions. Due to rising ambient air pollution in highly populated megacities such as Delhi, most Indian cities struggle to keep their inhabitants’ air livable. In the past three decades, Delhi’s population has grown by a factor of four, but the city’s motor vehicle population has increased by 28 [14]. There are currently more than 11.4 million registered automobiles in Delhi [15,16]. Transportation emissions have recently drawn much attention since many people are exposed to exhaust gases and other particles from moving vehicles. The contribution of transportation is believed to be as high as 45 percent in Delhi’s air pollution [17]. Private cars and two-wheelers make up most of the Delhi transportation sector’s emissions [18]. PM_2.5_ concentrations in Delhi vary throughout the day, with the highest levels during the evening rush hours and night [19]. The average contribution of the transportation sector to PM_2.5_ concentrations ranges from 17 to 28 percent, making it the main contributor to the city’s poor air quality [20]. Studies have indicated that the mortality burden of PM_2.5_ in Delhi can significantly decrease if exposure levels are reduced below the present levels [21]. Several sustainable national policies have been implemented to reduce transportation sector emissions and Delhi-specific pollution control measures [22]. However, Delhi’s air quality has not improved and has become a severe public health concern. Sustainable urban transportation policies may decrease ambient air pollution in New Delhi by putting it in place [23].

The commuters in Delhi are forced to use individualized modes of transportation due to the lack of a satisfactory alternative public transportation system [14]. As a result, the vast majority of adults were physically sedentary. The total physical activity in Delhi is estimated to be about 400.3 min per week for men and 265.3 min per week for women [4]. Being a low-emission and space-efficient mode of transportation, active transportation (mostly walking and bicycling) has grown in popularity in the transportation and environmental sectors in India. NMT or active transportation can provide both transportation (access to goods and activities) and recreation, while users may view a specific journey to serve both aims.

### 1.3. Co-Benefits of Nonmotorized and Active Transport

Co-benefits have been widely advocated in tandem with mitigation measures for greenhouse gas (GHG) reduction. Especially in developing nations such as India, where urbanization and climate change adaptation are all significant challenges, the health and economic co-benefits of nonmotorized transportation become particularly important [24]. Given its long-term benefits, NMT appears to be a feasible alternative to traditional fossil-fuel-based modes of public transportation in Indian cities. Walking and bicycling for transportation provide significant health benefits to users by increasing physical activity [25]. Benefits include improved accessibility, more affordable travel, less congestion, cheaper infrastructure costs for parking lots and roads, energy conservation, decreased air and noise pollution, and decreased accidents for other drivers [26]. Additionally, it is an affordable form of transportation for millions of people with low incomes, particularly in Delhi. 

The quantification of the co-benefits from climate change mitigation in the transportation sector has been investigated in previous studies, introducing the A–S–I (avoid–shift–improve) approach, assessing not just the avoided local air pollution and carbon emissions but also the advantages of improved air quality and its influence on public health. The results indicated a considerable decrease in CO_2_ and other local air pollutant emissions by 12.9 and 1.4 million tonnes, respectively, the prevention of almost 10,000 mortality cases, and a savings of more than USD 35 million by 2030 [27]. Pathak et al. assessed the sustainable and low-carbon mobility in India and quantified co-benefits, using the scenario analysis method, under different scenarios, including fuel transition, decarbonized electricity, modal change, and travel demand substitution, indicating that low-carbon transportation measures can offer significant development co-benefits that include a 74% reduction in NOx and an 83% reduction in PM_2.5_ from the passenger transportation sector compared with BAU in 2035 [28].

A review of thirty health impact assessment studies from Europe, the United States, Australia, and New Zealand concluded that health benefits from increased PA outweighed the adverse effects of traffic accidents and exposure to air pollution by a large margin. It is estimated that an increase in the median daily walking and bicycling time from 4 to 22 min can reduce 14% of GHG emissions in California, which results in decreasing the corresponding burden of cardiovascular disease and diabetes and could avoid 32,466 disability-adjusted life years (DALYs) [29]. Increased physical activity and reduced local air pollution from vehicle emissions can save 122 lives and net savings of about USD 200 million annually in New Zealand [30]. A study in Adelaide, Australia [31], showed that increased cycling, public, and active transportation could reduce road traffic-related CO_2_ emissions annually, ranging from 191,313 to 954,503 million tons and 160 to 542 deaths. Therefore, 2113 to 7674 DALYs could be avoided due to improved air quality, increased physical activity, and avoided traffic injury. Another study conducted in six European cities has shown that increases in cycling and walking trips to 35% and 50% of total city trips, respectively, will cut GHG emissions in the six cities by 1139 to 26,423 metric tons per year, including varying degrees of health benefits [32]. A recent study has indicated cost savings of 15 billion euros per year in Europe for a 10% shift to active mobility modes [33].

As transportation-related emissions rise in Delhi, NMT is becoming increasingly popular among policymakers and environmentalists as a practical substitute for motorized transportation. Studies have addressed the significant impacts of improving bicycle and bus infrastructure on lowering CO_2_ emissions [34] and increasing PA in 5% of the population [35]. Using the travel demand and health impact modeling approach, a study showed that active transportation can reduce the annual health burden (90,000 DALYs) in India [36]. Another study in India assessed alternative scenarios, including increased active travel, lower-carbon-emission motor vehicles, and a combination of the two, utilizing comparative risk assessment techniques [37]. The results indicated that the most significant advantages would come from combining active transportation with low-emission motor vehicles, which can reduce 12,995 DALYs in Delhi. 

### 1.4. What Will Be Elucidated in This Research: Model Development

**Figure 1:**
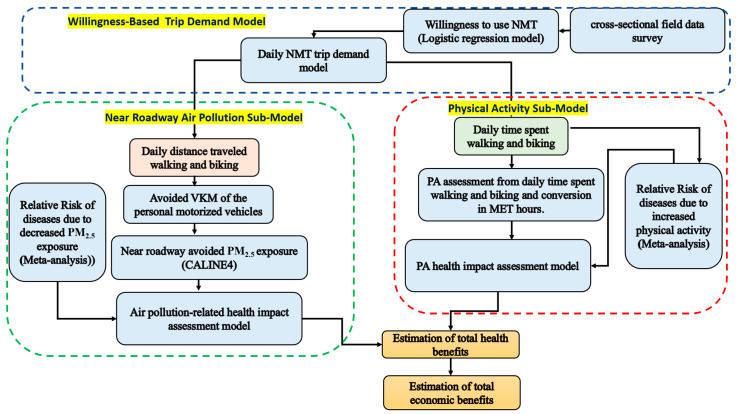
Integrated quantitative approach used in this study.

While there are inadequate studies in India conducted to estimate the co-benefits of NMT transportation, most of these studies have mainly focused on traffic injuries and air pollution and associated health benefits rather than the benefits of extra PA [38]. By developing an integrated quantitative assessment modeling framework of environmental–health–economic benefits (Figure 1), this paper seeks to quantify the expected health and economic co-benefits of both increased PA and improved air quality resulting from implementing the NMT policy in Delhi, India. As shown in Figure 1, the willingness-based trip demand model is used to estimate the total kilometers and time the inhabitants are willing to spend on NMT due to choosing to walk and bike instead of driving cars and motorized two-wheelers. The impact of the potential factors such as age, gender, education, income, and travel distance on the probability of the willingness to use NMT is analyzed, utilizing a logistic regression model on the collected data from a cross-sectional interview with 250 inhabitants in Delhi. The results of the trip demand model are used in two sub-models: (1) The physical activity sub-model converts the extra daily minutes of walking and cycling to metabolic equivalents (METs), which is further used in a health risk assessment to estimate the relationship between physical activity and the beneficial impact of lowering the occurrence of specific health outcomes in the selected traffic areas. (2) The near-road air pollution sub-model is used to calculate the potential reduction in motorized vehicle kilometers due to travel distance covered by walking and cycling and also its associated near-roadway-avoided PM_2.5_ exposure in 11 major districts in Delhi. The relationship between changes in PM_2.5_ concentrations and the occurrence of specific health outcomes in the chosen traffic areas is estimated, using the concentration–response function (CRF) for several diseases in order to establish a connection between the avoided exposure of PM_2.5_ and health benefits. The relative risk (RR) level, which predicts the likelihood of an adverse health outcome among the population exposed to a higher level of ambient air pollution than a lower level of ambient air pollution, served as the basis for the CRF coefficient values used in this study. The values of the RR used in the study were taken from a thorough meta-analysis of earlier research. In order to achieve this, a meta-analysis and a review of articles were carried out to determine the correlation between alterations in PM_2.5_ concentrations and alterations in the incidence of each health endpoint. The data for the avoided mortalities and morbidities from both sub-models are finally used to calculate the economic value of the avoided health burden from both the avoided emissions and increase in physical activity using the value of a statistical life (VSL) and cost of illness methods. The findings are generalized to the whole city of Delhi.

## 2. Materials and Methods (Model Development)

### 2.1. PA Estimation Model: Estimation of the Weekly Time Spent Walking and Cycling for Transportation

The counting model is first used to calculate the number of walks and bike trips an individual makes throughout an average day in order to estimate the weekly time spent walking and bicycling for transportation, which is expressed as follows [39]:

(1) $$M_{c,i}=N_{c,i}\times Pr_{c,i}\times t_{c,i}$$

where $M_{c,i}$ is the daily minutes spent traveling using mode *c* (walking or bicycle) for individual *i*; $N_{c,i}$ is the expected daily number of trips taken using mode c for individual *i;* $Pr_{c,i}$ is the probability that a trip taken by individual *i* using mode c and $t_{c,i}$ is the trip duration for a trip taken by individual *i* using mode c. $Pr_{c,i}$ expresses the willingness to adopt active transport (walking and bicycling), which can be further estimated by developing the following logistic regression model:

(2) $$Pr.\left(Y_{i}=1|X_{i}\right)=\frac{e^{X_{i}^{T}\beta }}{1+e^{X_{i}^{T}\beta }},\;\;\;\;\;i=1,2,\cdots ,n$$

In the above equation, $Y_{i}$ is the willingness to adopt active transport of the $i^{th}$ (i=1,2,⋯,n) individual. $X_{i}={\left(x_{i1},x_{i2},\cdots ,x_{ij},\cdots ,x_{ip}\right)}^{T}$ is the p×1 vector of covariates corresponding to the $i^{th}$ individual. $X_{i}$ represents the vector of independent regressors, including age, gender, education, income, and travel distance, which affect the individual’s willingness to use a walking or bicycle mode for transport purposes. Furthermore, $\beta ={\left(\beta_{1},\beta_{2},\cdots ,\beta_{j},\cdots ,\beta_{p}\right)}^{T}$ is the corresponding p×1 vector of regression coefficients, which can be estimated through the maximum likelihood estimation approach. The likelihood function of interest takes the following form:

(3) $$L\left(\beta |Y,X\right)=\prod_{i=1}^{n}\;{\left(Pr.\left(Y_{i}=1|X_{i}\right)\right)}^{Y_{i}}\;{\left(1-Pr.\left(Y_{i}=1|X_{i}\right)\right)}^{1-Y_{i}}.$$

The estimated coefficients of the above model are interpreted through odds ratios (ORs). The OR for the covariate $x_{ij}$ can be estimated from the estimates of regression coefficients as given below.

(4) $$OR\left(x_{ij}\right)=e^{\beta_{j}}$$

The daily PA can be calculated by combining the time spent cycling and walking multiplied by the intensity of each activity, as determined by metabolic equivalents (METs), which is explained as follows [39]:

(5) $$DPA_{i}=\frac{\left(MC_{walking\;,i}\times 3.5\right)+\left(Mc_{biking,\;i}\times 6.8\right)}{60\min/h}$$

where *DPA_i_* is the daily physical activity (from walking and cycling) for individual *i* in MET hours. For transportation, the MET values for walking and cycling are 3.5 and 6.8, respectively [40].

### 2.2. Near-Roadway-Avoided PM_2.5_ Exposure Model

The concentrations of air pollutants at particular locations are believed to represent population exposure levels. It is estimated that 55% of the population living within 500 m of major roads in Delhi are exposed to hazardous emissions from transportation, where PM_2.5_ is primarily produced by vehicle emissions [1]. It is crucial to use appropriate models with the vehicle and meteorological data to estimate near-roadway PM_2.5_ exposure, since monitoring PM_2.5_ concentrations cannot be achieved for all near-roadway regions. This research uses an air dispersion modeling tool called CALRoads View 6.5 (Lakes Environmental Software) to forecast how mobile sources affect air quality near roads and intersections. The CALINE-4 model is a fourth-generation line source air quality dispersion model that uses a mixing zone concept to characterize pollutant dispersion near roadways. Using the Gaussian dispersion methodology, the model predicts pollutant concentrations for receptors located within 150 m on either side of the roadways using input parameters such as site geometry and characteristics:

(6) $$C(x,y)=\frac{q\tau }{\pi \sigma_{z}u}\int_{y_{1}-y}^{y_{2}-y}\exp(\frac{-y^{2}}{2\sigma_{y}^{2}})dy$$

where *C* is the concentration (g/m^3^), *y_1_* and *y_2_* are the finite line source endpoint *y* coordinates (m), u is the wind speed (m/s), $\sigma_{y}$ and $\sigma_{z}$ are the horizontal and vertical Gaussian dispersion parameters which are a function of downwind distance *x* (m), *q* is the avoided PM_2.5_ emissions from replacing the distance traveled of the vehicle category τ (g/s), which can be calculated, as follows:

(7) $$q_{\tau }=(MC_{walking}K_{walking}+MC_{biking}K_{biking})EF_{\tau }$$

where $MC_{walking}$, $MC_{biking}$, $K_{walking}$ and $K_{biking}$ are the yearly time spent walking and cycling (s) and the average distance covered by walking and cycling in Delhi (km/s), respectively. $EF_{\tau }$ is the PM_2.5_ emission factor of the vehicle category τ (g/km) $MC_{walking}$, $MC_{biking}$ are calculated by Equation (1) and $K_{walking}$ and $K_{biking}$ are given values.

### 2.3. Health Impact Assessment Model

**Figure A2:**
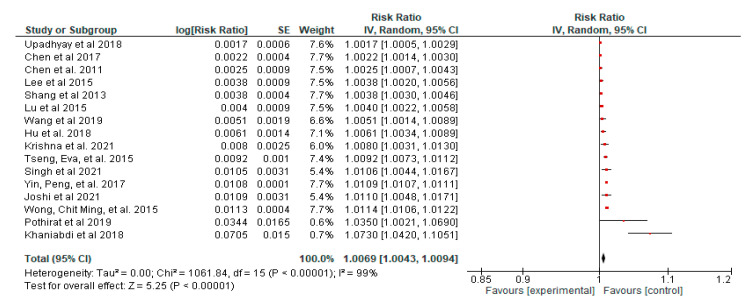
PM_2.5_-related all-cause mortality.

**Figure A3:**
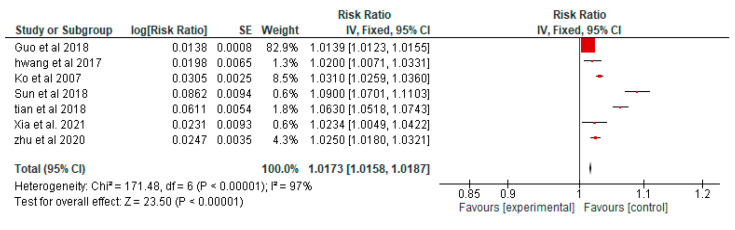
PM_2.5_-related COPD.

**Figure A4:**
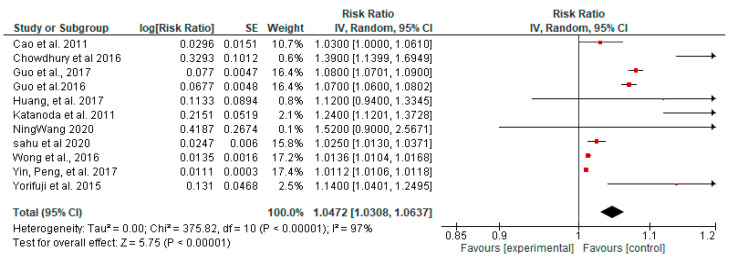
PM_2.5_-related lung cancer.

**Figure A5:**
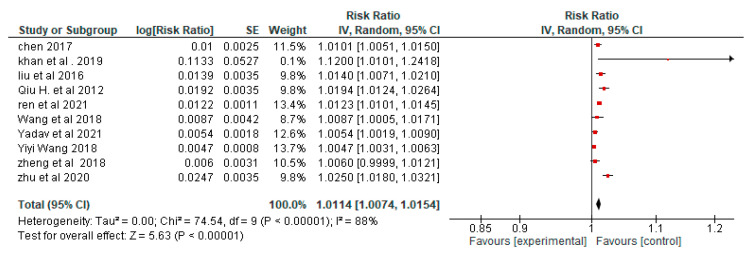
PM_2.5_-related respiratory-disease-related hospital admissions.

**Figure A6:**
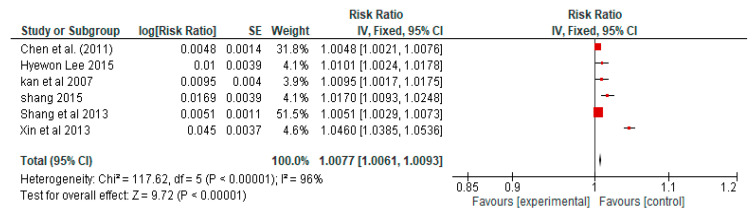
PM_2.5_-related respiratory mortality.

**Figure A7:**
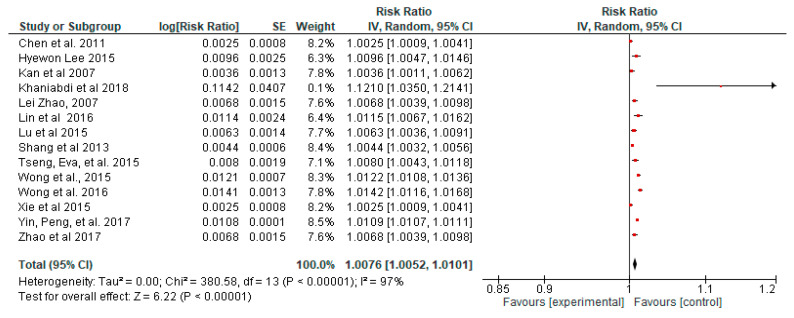
PM_2.5_-related cardiovascular mortality.

**Figure A8:**
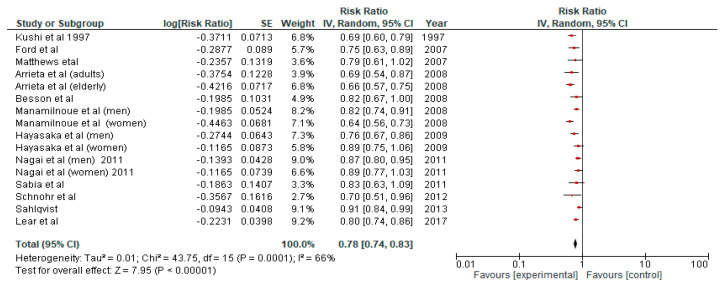
PA-related all-cause mortality.

**Figure A9:**
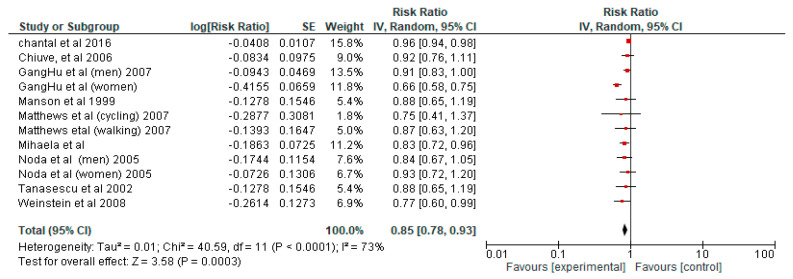
PA-related coronary heart diseases.

**Figure A10:**
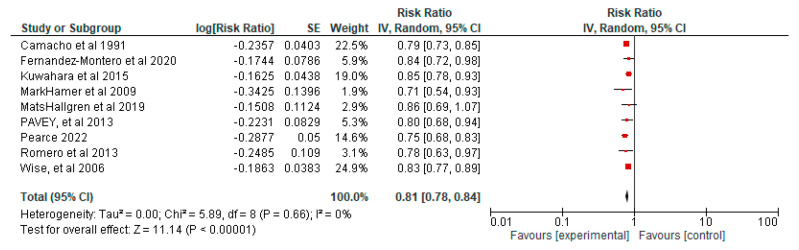
PA-related depression.

**Figure A11:**
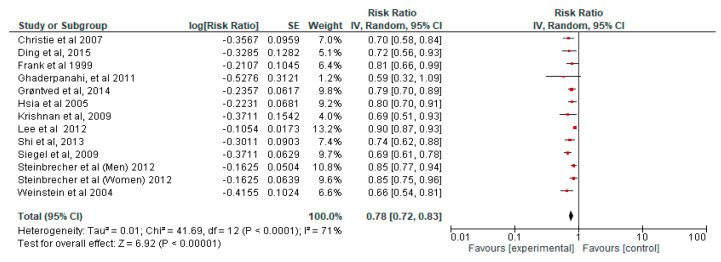
PA-related type 2 diabetes.

**Figure A12:**
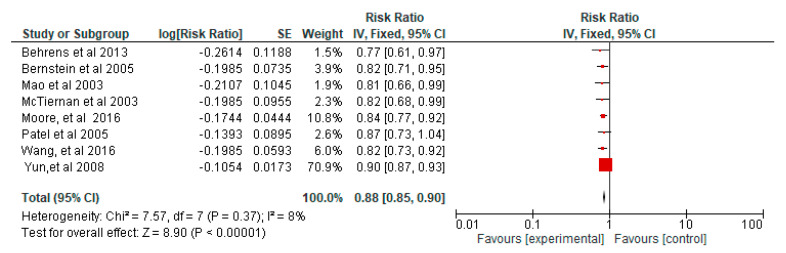
PA-related cancer.

Excess deaths or illnesses (EDI) caused by an increase in PM_2.5_ concentration can be estimated as follows [41]:

(8) EDI=PAF × I × P

where *I* is the annual baseline mortality rate, *P* is the total population, and *PAF* (population attributable fraction) indicates the percentage of illness burden attributable to pollution. The *PAF* can then be calculated using the formula below [42]:

(9) $$PAF=\frac{p(RR-1)}{p\left(RR-1\right)+1}$$

where *P* represents the population’s exposure rate. The values of *RR* for physical activity and near-roadway PM_2.5_ exposure are estimated in different ways. In order to accurately assess the correct values of RR, the health impact assessment requires highly aggregated epidemiological data. To this aim, in this study, a comprehensive meta-analysis was carried out based on a systematic quantitative review, where all directly relevant empirical data that satisfy the eligibility standards and criteria were gathered, combined, and statistically analyzed to produce a pooled estimate that was as close as possible to the *RR* values specified by Delhi. The following equation is used to estimate the final *RR* of willingness-based physical activity [39]:

(10) $$RR_{PA}=RR_{m}{}^{\left(\frac{DPA}{\alpha \;MET\;}\right)}$$

where $RR_{PA}$ is relative risk estimated from walking and bicycling physical activity; $RR_{m}$ is the relative risk estimated from the meta-analysis of the collected data from 61 studies on 5 physical activity-related health outcomes (all-cause mortalities, type 2 diabetes, coronary heart diseases, cancers, and depressive disorders) to estimate the dose–response function and PA-related morbidities and mortalities as the functions of physical transportation activity (Appendix A, Figure A2, Figure A3, Figure A4, Figure A5, Figure A6, Figure A7, Figure A8, Figure A9, Figure A10, Figure A11 and Figure A12). The meta-analysis was based on collecting data from selected studies with reported values of RR of moderate-intensity PA (α ranges from 3 to 6 h). The current global recommendation for adults’ physical activity is 150 min (α = 2.5 h) of moderate-intensity aerobic physical activity per week [43]. The pooled values of the RR collected from 64 studies related to total mortality, COPD, cardiovascular mortality, respiratory mortality, morbidity, and related hospital admission were used to calculate the relationship between a change in air pollutant concentration (in this study, PM_2.5_) and a change in health effects, i.e., concentration–response functions (CRFs):

(11) $$RR_{AP}=\exp\left[\beta \left(C-C_{0}\right)\right]$$

where $RR_{AP}$ is the relative risk estimated from the near-roadway-avoided PM_2.5_ exposure, and *β* is a coefficient that evaluates the response of a health outcome to a change in pollutant concentration. *C* and *C_o_* are the pollutant concentrations in the baseline and intervention scenarios. Table 1 represents the baseline incident rates of diseases in Delhi used in this study.

### 2.4. Economic Impact Assessment Model

The value of a statistical life (VSL) approach and the cost of an emergency room visit (ERV) were used in this study to calculate the mortality cost of PM_2.M_ exposure [41]. The cost of illness (COI) approach was used to determine the cost of treatment of coronary heart diseases, type 2 diabetes, cancer and depressive disorders, and hospital admissions due to respiratory diseases, COPD, respiratory mortality, cardiovascular mortality, and LC [44,45,46,47,48,49,50]. The values for the various health endpoints considered in this study are shown in Table 2.

## 3. Results and Discussion

### 3.1. Willingness of People in Delhi to Use NMT

A cross-sectional survey was conducted from 2 July to 8 July 2022, in Delhi, India, to estimate peoples’ willingness to accept active transportation as their usual travel mode. The survey encompassed different areas of Delhi, namely Ashok Vihar, Nand Nagri, Dilshad Garden, Jhilmil, and Seema Puri. About 250 inhabitants were physically interviewed during the survey, while 50 responses were collected online. People were physically approached and were asked questions during morning hours while leaving for work and during evening hours while returning, especially near bus stops, public parks, and pedestrian crossings. For the online survey, the questionnaires were shared with only those currently living in Delhi. The detailed questionnaire used in this research is described in Appendix B, Table A2.

The questionnaire, consisting of 11 questions, initiated the most important survey inquiry, whether the respondent was willing to use active transport in Delhi. Then, separate questions were asked for walking and bicycling. The later part of the questionnaire asked about the mandatory and nonmandatory travel modes of the inhabitants of Delhi, India, along with their preferable distance to be covered by walking and bicycling. Finally, the survey enquired about the respondents’ socio-economic condition (income and education) and demographic characteristics (age and gender). After discarding all the missing and erroneous data, a total of 283 respondents’ information was finally analyzed. All these variables are described in Appendix B, Table A1, which depicts that 30.35% and 38.52% of the respondents were willing to use walking and bicycling, respectively, as their regular transportation modes. All the selected covariates were categorized to distinguish the probability of willingness for the different categories of the corresponding predictors. Among the set of covariates, regular travel mode, monthly income, education level, and age were categorized into more than two categories. Such categorical variables require special attention, unlike continuous and binary predictors, which can be incorporated into the regression model without any modification. In the case of the covariates with more than two categories, one category needs to be considered as a reference category with which the remaining categories can be compared. This reference category is selected based on the purpose of comparison. Then, the remaining categories are transformed into binary variables and compared with the reference category of interest. The results of the model are presented in Table 3.

Regular travel mode, monthly income, and education level were found to be significant factors in the willingness to walk in Delhi, India. On the other hand, the LR model for the willingness to bicycle revealed significant associations between this willingness and regular travel mode, distance to cover, education level, gender, and age group, which are depicted in Table 3. The OR for private car users was 0.241, which is less than one. Hence, those who used private cars as their regular travel mode in Delhi had significantly (1−0.241)×100%=75.9% lower odds of preferring to walk than public transport users. Similarly, motorcycle users had 57.4% lower odds of willingness to walk regularly as a travel mode, compared with those who used public transport. A lower likelihood of bicycling was observed for both the regular users of private cars and motorcycles than those of public transport. Distance to cover could significantly influence only the willingness to bicycle. For longer distances (more than 2 km), people preferred (OR = 3.228 > 1 and *p* < 0.10) to use bicycling as a regular travel mode more than for shorter distances. Compared with the medium-income group, people with high incomes showed more tendency to choose walking as their travel mode in daily life. Both illiterate and highly educated people preferred walking more than those who completed their primary or secondary education. On the other hand, only illiterate people were more likely to bicycle than primary-to-secondary-educated ones. Both middle-aged and older people had a lower preference for bicycling than the younger group. On the other hand, compared with their female counterparts, males had a higher willingness to use bicycles in their daily lives. The *p*-values of the Hosmer–Lemeshow test for both the LR models were greater than 0.05, which indicates that the corresponding models did not provide a poor fit to the data [59]. 

Based on the NMT willingness analysis findings, the average per capita time spent on walking and bicycling were estimated as 11.1 and 2.3 min, respectively, which is equal to covering an extra walking and cycling distance of 1.18 km per day based on the average walking and cycling speed in Delhi. These results were further analyzed to evaluate the environmental and health impacts achieved by improving the infrastructure so that people in Delhi can adopt active transport as per their willingness. 

### 3.2. Near-Roadway-Avoided PM_2.5_ Exposure from Replacing the Distance Traveled by Private Vehicles with Walking and Cycling in Delhi

**Figure 2:**
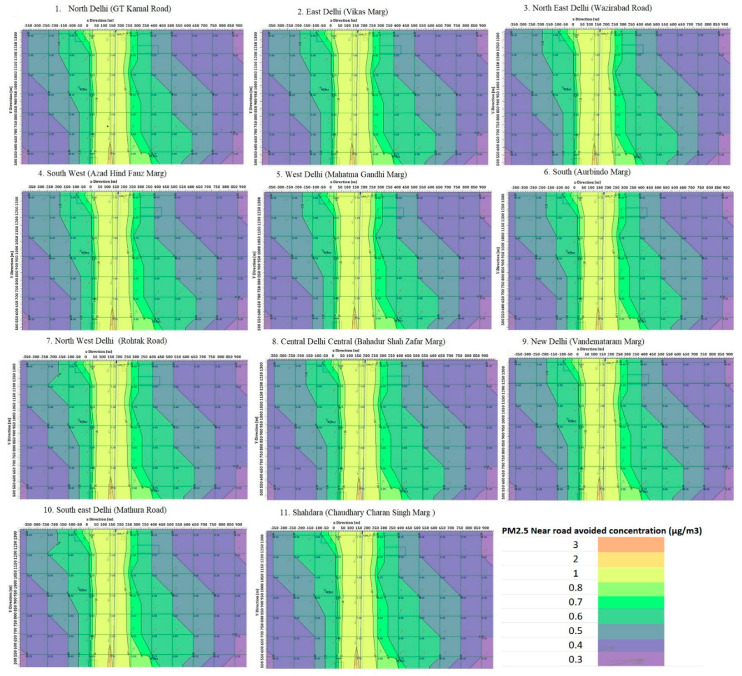
The heatmaps of avoided PM_2.5_ exposure in the selected traffic zones in different districts in Delhi.

The near-roadway concentration model described in Section 2.2 was applied to selected traffic zones (0.2 × 1 km^2^ area) in all 11 districts of Delhi while considering the population density in each district, in order to evaluate the impact of the avoided PM_2.5_ exposure from replacing the distance traveled by private vehicles with walking and cycling. The PM_2.5_ concentration was estimated by using the CalRoads software and was found to be 0.2 km downwind from vehicles to the near-roadway passengers. The heatmaps of the avoided PM_2.5_ exposure in the selected traffic zones in different districts in Delhi are shown in Figure 2. The per-hour-avoided vehicle kilometers (VKM) due to an increase in NMT and the average near-roadway-avoided PM_2.5_ exposure in the different districts in Delhi are shown in Table 4.

### 3.3. Public Health and Economic Co-Benefits from Replacing Distance Traveled by Private Vehicles with Walking and Cycling in Delhi

**Figure 3:**
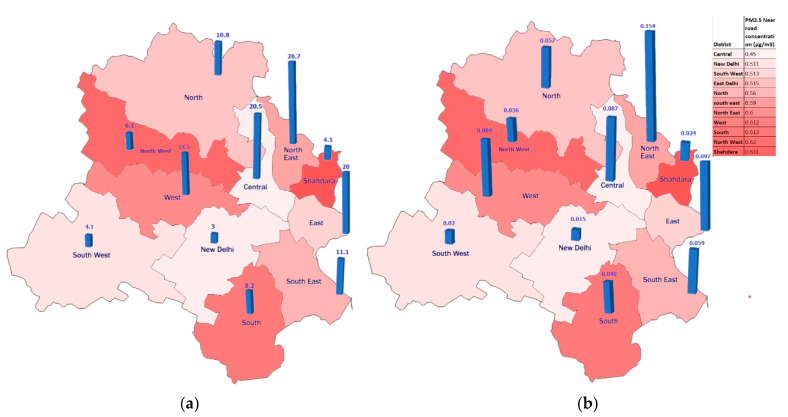
Estimated annual mortalities per km^2^ from (**a**) increased physical activity and (**b**) near-roadway-avoided PM_2.5_ exposure.

Figure 3a shows the avoided mortalities and morbidities related to increased PA per km^2^ in the different districts of Delhi. Northeast Delhi had the highest health benefits due to high population density, while the New Delhi district had the lowest health benefits due to less population density. More specifically, the NMT (walking and bicycling) use in areas with high population density, such as the northeast (36,155 people per kilometer), led to a more significant reduction in the number of morbidity and mortality cases. Figure 3b shows the avoided mortalities related to avoiding PM_2.5_ exposure near roadways in the various districts. The pooled values of the RR used in health impact assessment for both PA and near-roadway-avoided PM_2.5_ exposure are reported in Table 5 and Table 6. Table 7 includes the detailed avoided morbidities per km^2^ in the different districts of Delhi. As previously stated, the avoided health burden in each district depends on both traffic and the population density along the nearest roadway. Therefore, despite the fact that the southeast, Shahdara, and north districts had the highest avoided PM_2.5_ emission and exposure due to their heavy traffic conditions, the northeast and central districts had the highest expected avoided health cases due to higher near-roadway population densities. 

These findings were then generalized to the entire Delhi, taking into account the total area of the route network length of 16,200 km in Delhi [58,59,60,61,62,63]. The total avoided CO_2_ emissions and PM_2.5_ exposure were estimated at 121.5 kilotons per year and 138.9 tons per year, respectively (the emission factors and avoided VKM are given in Appendix C, Table A3). Finally, the total avoided health and economic impacts of physical activity and the associated reduction in near-roadway PM_2.5_ exposure are given in Table 8.

The results indicate that compared with the near-roadway-avoided PM_2.5_ exposure, increased PA had a higher impact on preventing annual mortalities and morbidities in the different districts of Delhi. This can be attributed to the higher values of the relative risks of PA, leading to higher health impacts. In addition, health benefits were seen as more prominent in the districts where the population density is higher, both in terms of increased PA and decreased air pollution. These results emphasize that an increase of 11.2 min of physical activity or 1.18 km of extra distance walked per day can significantly reduce noncommunicable diseases related to mortalities and morbidities in Delhi. The findings from this study are comparable with those of previous studies, which have predicted significant health and economic benefits of NMT policy implementation in India and other countries, emphasizing that the health benefits of active transport can outweigh the costs of NMT infrastructure requirements [63]. Similar studies in India have shown that active transportation can lower India’s health burden by 90,000 DALYs annually [35], and integrating active transportation with low-emission vehicles can save 12,995 DALYs in Delhi [36]. While considering economic impacts, a 10% shift to active mobility options has been indicated to save 15 billion euros annually in Europe [32].

## 4. Conclusions

Sustainable transportation can be integrated with development objectives such as health and well-being, clean energy, and sustainable cities. Climate change strategies are crucial, especially in developing nations such as India. In order to support the actions to mitigate climate change and other human development goals, it is essential to identify concrete co-benefits. Due to their lower carbon footprint and significant economic advantages in terms of preventing health effects, NMT can be a crucial part of such a strategy in Delhi. It can lower individual healthcare and transportation costs while enhancing public health and the environment. To this aim, this study introduced the integrated environmental, health, and economic benefits of implementing the NMT scenario in Delhi based on the willingness to walk and bike. 

The results revealed that increased physical activity and avoiding exposure to PM_2.5_ near roadways are expected to reduce the mortality rate by 40,018 cases in addition to reducing other morbidities, as indicated in this study, while physical activity was revealed to play a significant role in reducing mortalities and morbidities. The associated cost savings from morbidities and mortalities were estimated to be approximately USD 4869.8 million annually, which will positively impact Delhi’s local government’s finances. On the other hand, the local government may face difficulties and high costs when creating NMT facilities in Delhi due to the need to build and improve bike lanes, paths, and crosswalks as well as design safer roads for NMT transportation. However, massive savings in annual health costs may outweigh investments in infrastructure in just a few years.

## Figures and Tables

**Table 1:** Baseline incident rates of diseases in India (Delhi).

Mortality/Morbidity	Baseline Incidence per 100,000
Total mortality ^1^	1013
COPD prevalence rate ^2^	818
Type 2 diabetes prevalence rate ^2^	784
Respiratory disease rate ^2^	608
Depressive disorder rate ^2^	633
Diabetes mortality rate ^2^	18
Ischemic/Coronary heart disease prevalence rate ^2^	2321
Cardiovascular mortality ^1^	497
Respiratory mortality ^1^	66
Cancer prevalence rate ^4^	98.8
Lung cancer prevalence rate (LC) ^3^	9.06

^1^ From [38,39]. ^2^ Form [44]. ^3^ From [45]. ^4^ From [46].

**Table 2:** Values of the health endpoints used in this study.

Health Endpoint	Valuation (USD) with a Yearly 10% Increase in the Cost of Treatment	Method of Cost Calculation
Total mortality	279,411	VSL approach [41]
COPD hospital admissions	345	CoI approach [51]
Respiratory disease hospital admissions	80	CoI approach [51]
LC	1886	CoI approach [52]
Type 2 diabetes	23,862	[52,53,54]
Coronary heart diseases	3842	[55]
Depression	110	[55,56,57]
Cancer	1065	[58]

**Table 3:** Estimated coefficients $\hat{\beta }$ obtained from LR models along with OR and *p*-value ^1^.

Covariates	Willingness to Walk			Willingness to Use Bicycle		
	$\hat{\beta }$	OR	*p*-Value	$\hat{\beta }$	OR	*p*-Value
Intercept	−0.328	0.720	0.637	0.342	1.408	0.696
Regular travel mode						
Public transport	(RC ^2^)	(RC)	(RC)	(RC)	(RC)	(RC)
Private car	−1.421	0.241	<0.001	−0.740	0.477	0.035
Motorcycle	−0.853	0.426	0.018	−0.692	0.501	0.042
Distance to cover						
Short distance	(RC)	(RC)	(RC)	(RC)	(RC)	(RC)
Long distance	0.102	1.107	0.741	1.172	3.228	0.066
Monthly Income						
Low	0.147	1.158	0.716	−0.017	0.983	0.963
Medium	(RC)	(RC)	(RC)	(RC)	(RC)	(RC)
High	1.482	4.402	0.005	0.603	1.828	0.242
Education level						
Illiterate	1.261	3.529	0.023	1.089	2.971	0.041
Primary to secondary	(RC)	(RC)	(RC)	(RC)	(RC)	(RC)
Higher education	0.779	2.179	0.051	0.321	1.379	0.351
Age group						
Young	(RC)	(RC)	(RC)	(RC)	(RC)	(RC)
Middle-aged	−1.011	0.364	0.102	−2.233	0.107	0.002
Old	−0.961	0.383	0.193	−2.925	0.054	<0.001
Gender						
Male	0.371	1.449	0.264	0.557	1.745	0.076
Female	(RC)	(RC)	(RC)	(RC)	(RC)	(RC)
$\mathrm{McFadden}’s\;R^{2}$	0.112			0.100		
$\mathrm{Cox}-\mathrm{Snell}\;R^{2}$	0.128			0.120		
$\mathrm{Tjur}’s\;R^{2}$	0.140			0.124		
*p*-value of the Hosmer–Lemeshow test	0.66 (>0.05)			0.19 (>0.05)		

^1^ Obtained from the Wald test; ^2^ reference category.

**Table 4:** Per-hour-avoided VKM and average near-roadway-avoided PM_2.5_ exposure in different districts in Delhi.

District	Traffic Zone	Per-Hour-Avoided VKM	Near-Roadway-Avoided Concentration (µg/m^3^)
1. Northwest	Rohtak Road	261.3	0.62
2. South	Aurbindo Marg	235.3	0.62
3. West	Mahatma Gandhi Marg	232.9	0.62
4. Southwest	Azad Hind Fauz Marg	172.2	0.52
5. Northeast	Wazirabad Road	226.3	0.60
6. East	Vikas Marg	167.7	0.52
7. North	GT Karnal Road	200.9	0.56
8. Central	Bahadur Shah Zafar Marg	69.6	0.45
9. New Delhi	Vandemataram Marg	173.1	0.52
10. Southeast	Mathura Road	291.7	0.59
11. Shahdara	Chaudhary Charan Singh Marg (AV)	322.8	0.64

**Table 5:** RR values per moderate PA extracted from the meta-analysis.

	Avoided All-Cause Mortality	Coronary Heart Diseases	Depression	Diabetes	Cancer
Pooled value of RR with 95% CI	0.78	0.85	0.81	0.81	0.88

**Table 6:** RR values per 10 μg reduction in PM_2.5_ concentration extracted from the meta-analysis.

	Avoided All-Cause Mortality	Respiratory Mortality	Respiratory-Disease-Related Hospital Admissions	COPD	Cardiovascular Mortality	LC
Pooled value of RR with 95% CI	1.0069	1.0077	1.0114	1.0173	1.0076	1.0472

**Table 7:** Avoided morbidities (1 km^2^) with increased physical activity decreased near-roadway PM_2.5_ exposure.

District	CVD	Depression	Type 2 Diabetes	Cancer	COPD	LC	HA	RM	CVM
Northwest	13.93	4.98	6.16	0.46	0.004	0.001	0.045	0.003	0.018
South	18.67	6.67	8.26	0.62	0.005	0.001	0.059	0.004	0.024
West	33.03	11.8	14.62	1.09	0.009	0.001	0.104	0.006	0.041
Southwest	9.19	3.28	4.07	0.3	0.002	0.001	0.025	0.002	0.01
Northeast	61.05	21.82	27.02	2.02	0.016	0.002	0.192	0.011	0.076
East	45.81	16.37	20.28	1.52	0.01	0.001	0.121	0.007	0.048
North	24.58	8.78	10.88	0.81	0.006	0.001	0.071	0.004	0.028
Central	46.82	16.73	20.72	1.55	0.009	0.001	0.109	0.006	0.043
New Delhi	6.85	2.44	3.03	0.22	0.002	0.001	0.018	0.001	0.008
Southeast	25.33	9.05	11.21	0.84	0.006	0.001	0.073	0.004	0.029
Shahdara	9.19	3.28	4.07	0.3	0.003	0.001	0.03	0.002	0.012

**Table 8:** Total avoided mortality, morbidity, and economic impacts of improved PA and near-roadway-avoided PM_2.5_ exposure resulting from implementing NMT in Delhi.

Mortality	Morbidities							Economic Impacts
All-Cause Mortality (PA and AP)	CHD	Depression	Type 2 Diabetes	Cancer	COPD	LC	HA	Avoided Cost (Million USD)
17529	39,707	14,190	17,575	1319	20	1.8	248	4869.8

**Figure A1:**
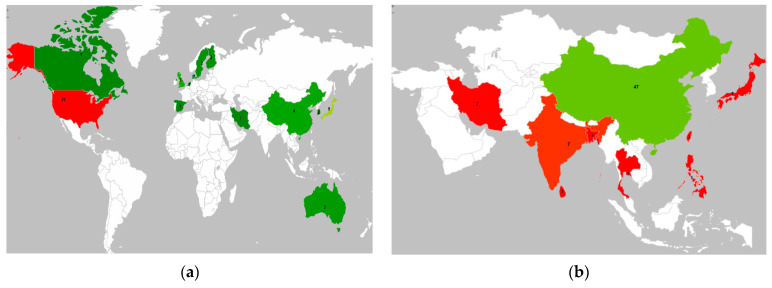
The geographical distribution of the studies that were chosen for the RR meta-analysis: (**a**) physical activity and health impacts; (**b**) PM_2.5_ and health impacts.

## Data Availability

Not applicable.

## Appendix A

This meta-analysis included 64 + 61 studies based on the inclusion criteria using the RAVMAN (5.4.1 version) software. The following tables show the pooled risk ratio (RR) for total mortality (PM_2.5_), COPD, cardiovascular mortality, respiratory mortality, LC, and respiratory morbidities related to hospital admissions, cancer, type 2 diabetes, depression, total mortalities (PA), and coronary heart diseases from 64 studies related to PM_2.5_ and health impacts, and 61 studies that were included in the meta-analysis.

## Appendix B

The questionnaire used for the estimation of willingness for NMT in Delhi.

**Table A1:** Characterization of selected variables along with descriptive statistics.

Variables	Categories	Characteristics	Frequencies (%)
Willingness to walk	Yes No	Willed to use walking as a regular travel mode Unwilled to use walking as a regular travel mode	85 (30.35%) 198 (69.95%)
Willingness to bicycle	Yes No	Willed to use bicycling as a regular travel mode Unwilled to use bicycling as a regular travel mode	109 (38.52%) 174 (68.48%)
Regular travel mode	Public transport Private car Motorcycle	Used public transport as a mandatory travel mode Used private car as a mandatory travel mode Used motorcycle as a mandatory travel mode	99 (34.98%) 96 (33.92%) 88 (31.10%)
Distance to cover by walking	Short distance Long distance	Preferred to walk for 1–2 km of distance Preferred to walk for more than 2 km of distance	96 (33.92%) 187 (66.08%)
Distance to cover by bicycling	Short distance Long distance	Preferred to use the bicycle for 1–2 km of distance Preferred to use the bicycle for more than 2 km of distance	19 (6.71%) 264 (93.29%)
Monthly income	Low income Medium income High income	Monthly income of below 15,000 INR Monthly income of between 15,000 and 75,000 INR Monthly income of above 75,000 INR	90 (31.80%) 172 (60.78%) 21 (7.42%)
Education level	Illiterate Primary to secondary High	No education Studied up to school Graduated or higher studies	25 (8.83%) 91 (32.16%) 167 (59.01%)
Age group	Young Middle-aged Old	Aged between 10 and 18 years Aged between 18 and 45 years Aged above 45 years	18 (6.36%) 224 (79.15%) 41 (14.49%)
Gender	Male Female	Male respondents Female respondents	200 (70.67%) 83 (29.33%)

**Table A2:** Detailed questionnaire used in this survey.

Question	Response
1. Willing to use NM modes (bicycle)	Yes No Already using NM modes
2. Willing to use NM modes (walking)	Yes No Already using NM modes
3. Mandatory travel mode to work, school, business	(Walk/Bicycle) motorcycle personal vehicle/car Public transport
4. Nonmandatory travel mode to shopping, personal, family, etc.	(Walk/Bicycle) motorcycle personal vehicle/car Public transport
5. Age-wise distribution	Below 18years 18–25 years 25–45 years 45–55 years 55–65 years
6. Gender-wise distribution	Female Male
7. Occupation level	Student Support Middle Higher
8. Monthly income level	below 15 k 15k to 30 k 30k to 50 k 50k to 75 k 75k to 100 K above 100 K
9. Education level	Nil Only school Graduation or Higher.
10. For which distance you prefer bicycles/walking as your transport mode?	1–2 Km 2–3 Km 3–4 km 4–5 Km 5–6 Km
Personal vehicle ownership	motorcycle personal vehicle/car Personal cycle None
11. What are the main barriers towards walking and cycling in urban area?	Few cycle lanes and bike parking areas Safety: no separate road space for cycling and walking. Inadequate bicycle rental and bike-sharing systems No combination of cycling and rapid bus transit or rail systems) No cycling/Walking culture Other

## Appendix C

**Table A3:** Total annual avoided PM_2.5_ and CO_2_ in Delhi based on reduced VKM [17,62,63,64,65,66,67].

Vehicle Type	Total VKM Per Year (Millions)	Reduced VKM (Millions)	Reduced PM_2.5_ (Tons)	Reduced CO_2_ (Kio Tons)
Car	6358.93	426.69	64.01	73.8
Motorcycle (2-wheeler)	11,624.63	780.02	56.95	35.6
Three wheelers	2430.98	163.12	17.95	12.1
Total	20,414.53	1369.82	138.9	121.5
